# The Impact of Bullying Victimization on Short Video Addiction in Adolescents: The Role of Emotional Distress and Neural Mechanisms

**DOI:** 10.1111/adb.70038

**Published:** 2025-04-21

**Authors:** Qiong Yao, Wenwei Zhu, Yuanyuan Gao, Jinlian Wang, Chang Liu, Guang Zhao, Qiang Wang

**Affiliations:** ^1^ School of Educational and Psychological Science Hefei Normal University Hefei China; ^2^ Key Laboratory of Philosophy and Social Science of Anhui Province on Adolescent Mental Health and Crisis Intelligence Intervention Hefei China; ^3^ School of Psychology South China Normal University Guangzhou China; ^4^ Faculty of Psychology Tianjin Normal University Tianjin China

**Keywords:** adolescents, bullying, intersubject representational similarity analysis, negative emotion, short‐video addiction

## Abstract

Short‐video addiction (SVA) has become a growing concern among adolescents. Bullying victimization (BV) is considered a significant factor contributing to it, yet its relationship with SVA remains underexplored. This study investigated the role of BV in SVA, examining developmental and psychological pathways across middle school students (MSS; *n* = 1269), college students (CS; *n* = 1615) and a replicated college sample (RCS; *n* = 112). Descriptive statistics revealed significant correlations between SVA and BV, including subdimensions such as verbal, physical and relational bullying, as well as negative affect (NA). Mediation analyses showed that NA partially mediated the relationship between BV and SVA across both MSS and CS groups, although mediation effects were absent in addicted subgroups, highlighting differing psychological pathways between addicted and nonaddicted populations. Neuroimaging analyses in the RCS sample identified spontaneous functional brain activity linked to SVA in the inferior temporal gyrus (ITG) and parahippocampal gyrus (PHG), with intersubject representational similarity analyses (IS‐RSA) further associating PHG and dorsomedial prefrontal cortex (DMPFC) activity patterns with intersubject variations in SVA. These findings underscore bullying victimization as a critical predictor of short video addiction, mediated by NA in nonaddicted groups, and illuminate spontaneous brain activity patterns associated with addiction.

## Graphical abstract

This study examined how bullying victimization contributes to short‐video addiction (SVA) among adolescents. Emotional distress was found to mediate this relationship in nonaddicted groups. Neuroimaging results revealed brain activity patterns associated with SVA in regions including the inferior temporal gyrus and dorsomedial prefrontal cortex.

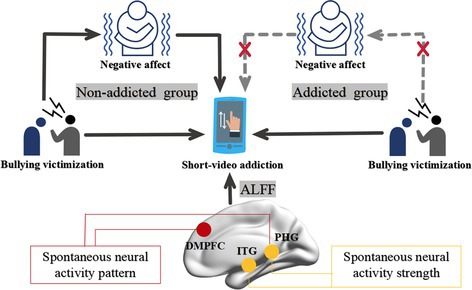

## 1 Introduction

Short‐format videos, characterized by their fragmented structure and diverse content, have become a widespread medium for individuals seeking to engage with digital content [1]. These videos are often utilized as a coping mechanism to manage life pressures and emotional distress [2]. However, excessive consumption of short‐form videos can lead to addictive behaviours [3]. Short‐video addiction (SVA), a subtype of Internet addiction, is defined as the compulsive or excessive use of short videos for the purpose of mood alteration [4]. Adolescents, in particular, are at heightened risk due to their undeveloped capacities for self‐regulation and impulse control. This age group is also undergoing a sensitive phase of psychological development, during which they are more susceptible to external stressors [5], further increasing the likelihood of short video addiction.

Bullying, which is defined as power imbalances, repetitive aggression and social exclusion [6], has been shown to exacerbate the risk of Internet addiction in adolescents [7]. According to the social compensation theory, individuals who experience negative life events, such as bullying, may turn to social media as a means of alleviating negative emotions [8]. In stressful situations, social media can provide a platform for seeking social support, fostering a sense of shared understanding [9] and facilitating access to potential solutions for emotional distress [10]. Therefore, adolescents who are victims of bullying may be more likely to use social media as a coping strategy, which can further contribute to addictive behaviours. Previous research has demonstrated that bullying victims are more likely to use platform like Instagram as an escape, leading to increase Instagram addiction [11]. Furthermore, both traditional bullying and cyberbullying have been found to predict problematic Internet use behaviours in adolescents [12]. Bullying also exerts a profound emotional toll on adolescents, often leading to anxiety, depression, loneliness and low self‐esteem [13]. These emotional challenges can have long‐lasting, impairing adolescents' mental health and social functioning.

Emotion regulation theory posits that individuals, particularly adolescents, are more likely to seek external methods of emotion regulation when confronted with negative emotions [14]. Due to their developing emotional regulation capacities, adolescents are particularly prone to using short‐video platforms for instant emotional gratification. The immediate entertainment and emotional feedback provided by these platforms meet their emotional needs in the short term. Research suggests that emotional regulation is a key motivator for short video use, particularly in response to negative emotions such as anxiety, depression and loneliness [15]. However, SVA should not be viewed as a healthy or effective form of emotional regulation. Rather than addressing the root causes of emotional distress, SVA reinforces a cycle of dependence on the platform, which exacerbates emotional issues over time [9].

Although SVA has garnered significant research attention, the psychological mechanisms underlying this addictive behaviour remain insufficiently understood. Existing studies have identified factors such as cognitive biases, personality traits and social influences as potential contributors to SVA [3]. However, there remains a gap in research regarding the influence of negative life events, such as bullying, on the development of SVA. Given the unique developmental characteristics of the adolescents, it is critical to explore the relationship between bullying, emotional distress and SVA. This study seeks to directly investigate whether bullying victimization is positively associated with SVA and whether negative emotions mediate this relationship.

Adolescents' psychological and cognitive development significantly influences their ability to cope with stressors like bullying. Middle school students, in the middle to late adolescent stage, show rapid development in brain regions related to reward processing (e.g., nucleus accumbens), whereas areas for emotion regulation and impulse control remain immature (e.g., prefrontal cortex) [16]. This makes them more vulnerable to mood fluctuations and social stress. College students, in the preadult stage, have more mature cognitive and emotion regulation abilities, with a fully developed prefrontal cortex (PFC) that aids in decision‐making and self‐control [17]. This maturity allows them to regulate emotions and cope with distress more rationally. This study compares middle school and college students to examine the psychological mechanisms behind bullying, negative emotions and short video addiction, with the goal of better understanding and developing effective intervention strategies.

To gain a deeper understanding of the neural mechanisms underlying SVA, an increasing number of studies have begun to investigate the relationship between addictive behaviours and brain activity. In particular, fMRI studies in adolescents with SVA have revealed disruptions in brain regions such as the prefrontal, occipital and parietal regions, which may impair cognitive control and reward evaluation [18]. Moreover, the reward pathways in the brain, including nucleus accumbens and caudate, are overactivated in individuals with SVA when watching short videos, leading to intense feelings of pleasure and satisfaction [18]. This overactivation weakens the brain's response to normal rewards, further reinforcing the addictive behaviour. The abnormal activation of the reward system observed in SVA shares similarities with patterns seen in substance addicts [19], suggesting that SVA may constitute a novel mental disorder. Given these parallel with substance addiction, further exploration of the neural substrates of SVA is warranted.

The amplitude of low‐frequency fluctuations (ALFF), a quantitative measure reflecting regional spontaneous activity intensity in blood‐oxygen‐level‐dependent (BOLD) signals [20], has demonstrated predictive validity for substance addiction susceptibility in clinical populations [21]. Intrinsic spontaneous neural fluctuations are thought to be associated with various addictive behaviours, including internet addiction and SVA [22]. Complementing this approach, intersubject representational similarity analysis (IS‐RSA) allows researchers to compare neural activity patterns across different individuals in response to the same stimuli or experiences, enabling the identification of common brain representations [23, 24]. Unlike traditional approaches, which may focus on average group activation or univariate analyses, IS‐RSA captures the variability in neural responses and highlights the commonalities and differences in brain processing across subjects. It facilitates the exploration of how brain activity corresponds to behaviour or emotional states across individuals. In addition, IS‐RSA can compare different types of representations (e.g., behavioural, neural or cognitive) across individuals, which is especially useful when integrating multimodal data. In this study, we compared behavioural (SVA) and neural (ALFF) representations to examine how the brain encodes and processes SVA across individuals, providing more accurate insights into the nature of the brain networks involved.

This study aims to (1) provide a comprehensive analysis of the relationship between bullying victimization, negative emotional states and short‐video addiction (SVA); (2) examine whether negative emotional states mediate the connection between bullying victimization and SVA; and (3) explore the neural substrates underlying SVA through the use of ALFF and IS‐RSA techniques.

## 2 Materials and Methods

### 2.1 Participants

Two datasets were utilized in this study. First, to examine the mechanism underlying SVA, data were collected from a total of 3038 students, comprising 1310 middle school students (MSS) and 1728 college students (CS). After excluding 154 participants due to incomplete (*n* = 79) or low‐quality data (*n* = 34), the final sample included 2884 (59.15% females, ages 12–24 years; MSS = 1269; CS = 1615). A chi‐square test indicated a significant difference in gender distribution between MSS and CS groups (*χ* ^2^ = 60.195, *p* < 0.001). Additionally, a separate group of 112 college students (68 males; ages 17–30; Mean age ± SD = 20.08 ± 2.20) were recruited, and resting‐state fMRI data were collected from these participants to investigate the neural substrate of SVA. For the MSS, they were required to sign the consent form after receiving the verbal consent from their parents via telephone. For the CS, written informed consent was obtained from each of them. This study was approved by the Institutional Review Board of Tianjin Normal University (IRB ID: XL2020‐27).

### 2.2 Measures and Questionnaires

#### 2.2.1 Short‐Video Addiction

SVA was assessed using the Smartphone Addiction Scale Short Revision (SAS‐SV), originally developed by Kwon et al. [25]. In this study, the term ‘mobile phone usage’ was substituted with ‘short‐video app use (e.g., Tik Tok, Kwai and Douyin)’ in each item, as suggested by Chao et al. [26]. The scale employed a 6‐point continuum, with statements representing varying degrees of SVA. A gender‐specific cutoff score was applied to distinguish between normal users (or nonaddicted group) and those at risk for addiction (or addicted group): 33 for females and 31 for males. The internal consistency of the scale was high, with a Cronbach's α of 0.897.

#### 2.2.2 Bullying Victimization

Bullying victimization was measured using the Delaware Bullying Victimization Scale‐student (DBVS‐S) [27]. The DBVS‐S is a 14‐item instrument that assesses four dimensions of bullying: (i) verbal bullying, (ii) physical bullying, (ii) relational bullying and (iv) cyberbullying. A 6‐point continuum was used to capture varying degrees of bullying victimization. In this study, the cyberbullying dimension was excluded due to ongoing debates about whether it should be considered part of the same construct as other forms of bullying [28]. Additionally, cyberbullying typically occurs outside the school context, weakening its connection to the campus environment [29]. The scale demonstrated strong internal consistency, with a Cronbach's a of 0.946.

#### 2.2.3 Negative Affect

The Positive and Negative Affect Schedule (PANAS) was used to measure the experience of positive and negative affect [30]. Participants rated the extent to which they experienced various feelings and emotions over the past month, with separate scale for (i) positive affect and (ii) negative affect. The internal consistency of the scale was high, with a Cronbach's α of 0.841.

### 2.3 Brain Imaging Data Acquisition

Rs‐fMRI scans were conducted using a Siemens 3 Tesla Prisma scanner with a 64‐channel head coil at Tianjin Normal University's MRI Center. Functional images were acquired using GRE‐EPI sequences with the following parameters: TR = 2000 ms, TE = 30 ms, multiband factor = 2, flip angle = 90°, FOV = 224 × 224 mm^2^, slice thickness = 2 mm, voxel size = 2 × 2 × 2 mm^3^. The scan consisted of 300 volumes, lasting 10 min and 13 s. Subjects were instructed to relax, keep their eyes closed and stay awake.

### 2.4 Resting‐State fMRI Preprocessing

Resting‐state fMRI data were preprocessed using fMRIPrep 23.1.3 [31], with the Nipype‐based pipeline [32]. The pipeline included (1) susceptibility distortion correction via fieldmap‐based (FMB) method; (2) coregistration of BOLD reference image to T1w anatomical image with boundary‐based registration in FreeSurfer's bbregister; (3) motion correction using MCFLIRT [33]; (4) slice‐timing correction with AFNI's 3dTshift [34]; (5) processing of the T1w images using the ‘recon all’ pipeline in FreeSurfer [35]; and (6) resampling for motion, susceptibility distortion and coregistration. The preprocessed data were further processed with the eXtensible Connectivity Pipeline (XCP) [36], including removal of dummy scans, nuisance regression (36P strategy) and despiking with AFNI's 3dDespike. The data were denoised using linear regression in Nilearn 0.10.0 [37], band‐pass filtered (0.01–0.1 Hz) and recensored. Whole‐brain ALFF was computed by extracting the power spectrum within 0.01–0.1 Hz and calculating voxel‐wise ALFF maps [38]. These maps were smoothed (FWHM = 6.0 mm) for GLM analyses and kept unsmoothed for IS‐RSA.

### 2.5 Univariate and IS‐RSA Analyses

We investigated the associations between SVA and ALFF across the whole brain employing a mixed‐effects FLAME 1 model within FSL. Covariates included maternal and paternal education levels, age, gender and FD. Statistical significance was determined at the cluster level (*z* > 3.1, *p* < 0.01) using a family‐wise error rate of 0.05, with corrections for multiple comparisons applied through Gaussian Random Field Theory. Regions of interest (ROIs) were defined by selecting clusters that showed significant correlations with SVA.

We used IS‐RSA to investigate how brain functional patterns varied with intersubject differences in SVA. A dissimilarity matrix for each participant pair was created based on Euclidean distances across the 10 SVA scale items. ALFF pattern dissimilarities were computed using a representational similarity matrix for each brain parcel (*n* = 200), based on whole‐brain parcellation informed by Neurosynth meta‐analytic data (http://neurovault.org/images/39711). Pearson's correlation measured pairwise similarity between participants. We examined the correlation between brain parcel dissimilarities (1 − *r*) and behavioural dissimilarities using Pearson's correlation, focusing on the lower triangle of the matrices. Bonferroni correction was applied to adjust *p* values for multiple comparisons.

## 3 Results

### 3.1 Descriptive Statistics

Table 1 presents demographic information and each scale's scores, along with comparisons across three groups: middle school students (MSS; *n* = 1269), college students (CS; *n* = 1615) and replicated college samples (RCS; *n* = 112). In the MSS group, the scores of SVA ranged from 10 to 60 (M ± SD = 24.07 ± 10.45). Bullying victimization scores spanned from 4 to 32 across various subdimensions, including verbal (*M* ± SD = 9.67 ± 5.11), physical (*M* ± SD = 7.43 ± 3.87) and relational bullying (*M* ± SD = 8.25 ± 4.43). Significant gender differences were observed in SVA (*t*[1267] = −3.211, *p* = 0.001) and physical bullying (*t*[1267] = 3.095, *p* = 0.002). Additionally, SVA was positively correlated with bullying victimization (*r* = 0.363, *p* < 0.001), verbal (*r* = 0.338, *p* < 0.001), physical (*r* = 0.288, *p* < 0.001), relational bullying (*r* = 0.356, *p* < 0.001) and negative affect (*r* = 0.291, *p* < 0.001) (Table 2).

**TABLE 1:** Demographics and basic questionnaire scores.

Measures	MSS (*n* = 1269)	CS (*n* = 1615)	RCS (*n* = 112)	*t*/*χ* ^2^ (MSS vs. CS)	*p*
Gender (M/F)	620/649	558/1057	68/44		
SVA	24.07 ± 10.45	24.80 ± 8.77	32.29 ± 10.57	−2.03	0.042*
BV	25.35 ± 12.15	18.21 ± 7.87		19.06	< 0.001***
VB	9.67 ± 5.11	6.43 ± 3.06			
PB	7.43 ± 3.87	5.69 ± 2.39			
RB	8.25 ± 4.43	6.09 ± 2.83			
NA	27.63 ± 8.33	23.90 ± 7.02	24.77 ± 8.17	13.03	< 0.001***

Abbreviations: BV, bullying victimization; CS, college students; MSS, middle school students; NA, negative affect; PB, physical bullying; RB, relational bullying; RCS, replicated sample of college students; SVA, short‐video addiction; VB, verbal bullying.

* *p* < 0.05.

** *p* < 0.01.

*** *p* < 0.001.

**TABLE 2:** Pearson's correlation coefficients with SVA in MSS (*n* = 1269).

Variable	Gender	SVA	BV	VB	PB	RB	NA
Gender	—						
SVA	3.211***	—					
BV	0.682	0.363***	—				
VB	0.469	0.338***	0.927***	—			
PB	3.095**	0.288***	0.879***	0.725***	—		
RB	−1.374	0.356***	0.906***	0.757***	0.701***	—	
NA	−1.785	0.291***	0.390***	0.380***	0.308***	0.363***	—

*Note:* All correlations significant at *p* < 0.001.

Abbreviations: BV, bullying victimization; NA, negative affect; PB, physical bullying; RB, relational bullying; SVA, short‐video addiction; VB, verbal bullying.

*p < 0.05.**p < 0.01.***p < 0.001.

In the CS group, the scores of SVA ranged from 10 to 60 (M ± SD = 24.8 ± 8.77) and varied by the bullying victimization (*r* = 0.294, *p* < 0.001), verbal (*r* = 0.295, *p* < 0.001), physical (*r* = 0.259, *p* < 0.001), relational bullying (*r* = 0.280, *p* < 0.001) and negative affect (*r* = 0.373, *p* < 0.001). Pairwise correlations between SVA, bullying victimization (BV), verbal bullying (VB), physical bullying (PB), relational bullying (RB) and NA are shown in Table 3. These findings were consistent with those observed in the MSS group. Direct comparisons between MSS and CS further revealed significant differences in SVA (*t*_[2882]_ = −2.03, *p* = 0.042), bullying victimization (*t*_[2882]_ = 19.06, *p* < 0.001) and negative affect (*t*_[2882]_ = 13.03, *p* < 0.001). These findings suggest developmental differences across groups, particularly regarding SVA, bullying victimization and emotional experiences.

**TABLE 3:** Pearson's correlation coefficients with SVA in CS (*n* = 1615).

Variable	SVA	BV	VB	PB	RB	NA
SVA	—					
BV	0.294***	—				
VB	0.295***	0.953***	—			
PB	0.259***	0.938***	0.833***	—		
RB	0.280***	0.958***	0.865***	0.865***	—	
NA	0.373***	0.400***	0.411***	0.338***	0.383***	—

*Note:* All correlations significant at *p* < 0.001.

Abbreviations: BV, bullying victimization; NA, negative affect; PB, physical bullying; RB, relational bullying; SVA, short‐video addiction; VB, verbal bullying.

*** *p* < 0.001.

Additionally, similar behavioural patterns were observed in the replicated college sample (RCS; *n* = 112), although with some distinct characteristics. Notably, bullying victimization scores were not collected in this sample, yet a significant correlation was still found between SVA and NA (*r* = 0.341, *p* < 0.001).

### 3.2 Common Method Biases

Harman's single‐factor test was applied to assess common method bias [39], with all items subjected to unrotated exploratory factor analysis across all datasets. The results indicated that seven common factors with eigenvalues greater than 1 were extracted from the factor analysis in the MSS dataset. The first factor accounted for 24.35% of the total variance, which was below the critical threshold of 40%, suggesting that common method bias was not a significant concern in the MSS dataset. Similarly, no evidence of common method biases was found in the CS datasets, where five common factors with eigenvalues greater than 1 were extracted, with the first factor accounted for 28.49% of the total variance.

### 3.3 NA Mediated the Relationship Between BV and SVA in MSS

**FIGURE 1:**
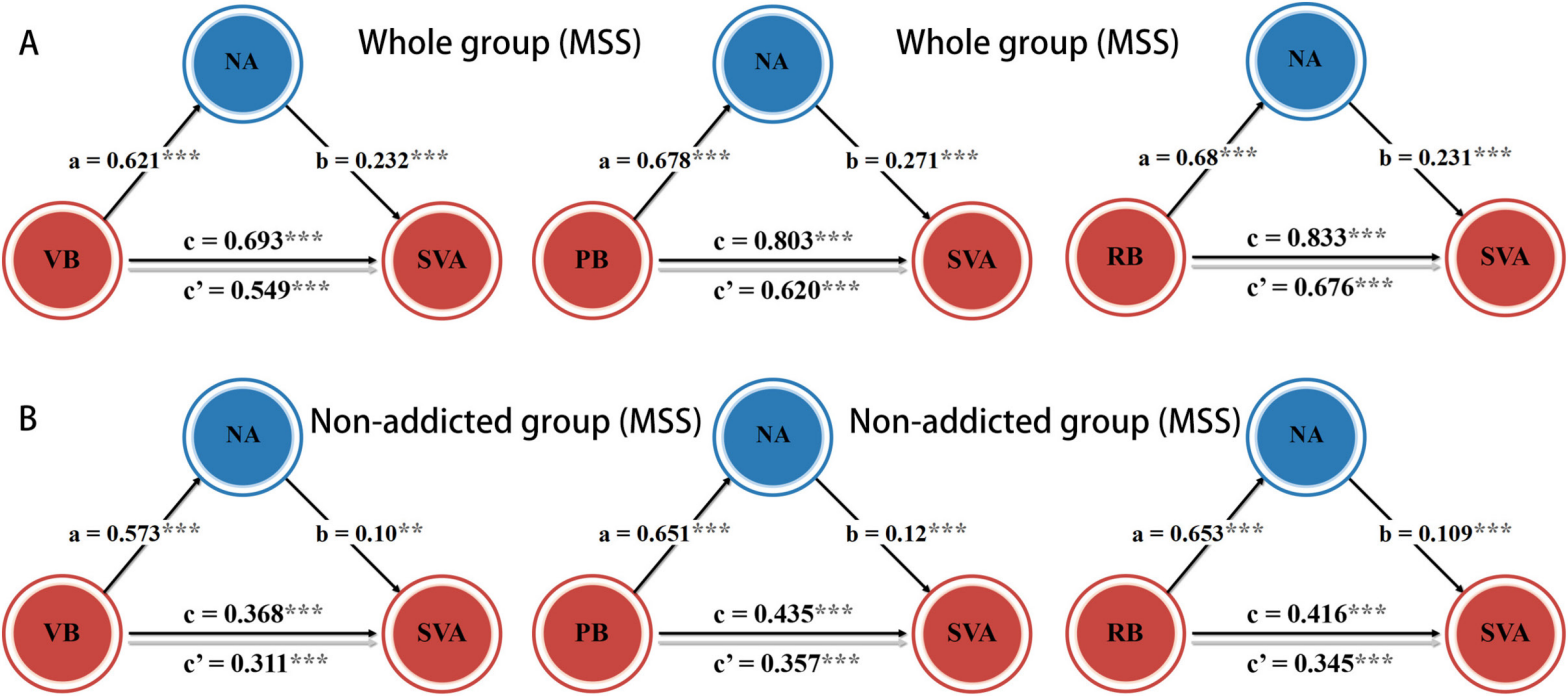
NA mediated the associations between bullying victimization and SVA in MSS. The impacts of various subdimensions of bullying victimization on SVA were mediated by the NA in the whole sample of MSS (*n* = 1269; A) and nonaddicted subgroup of MSS (*n* = 988; B). Abbreviations: MSS, middle school students; NA, negative affect; PB, physical bullying; RB, relational bullying; SVA, short‐video addiction; VB, verbal bullying.

NA was found to partially mediate the relationship between BV on SAVS (indirect effects = 0.057, 95% CI = [0.038, 0.081], mediation effect accounted for 18.15%). Furthermore, NA also mediated the associations between SVA and the subdimensions of bullying victimization, including verbal (indirect effects = 0.144, 95% CI = [0.097, 0.018], mediation effect accounted for 20.78%), physical (indirect effects = 0.183, 95% CI = [0.127, 0.249], mediation effect accounted for 22.79%) and relational bullying (indirect effects = 0.157, 95% CI = [0.038, 0.081], mediation effect accounted for 18.85%) (Figure 1A).

In the nonaddicted group (*n* = 988), we observed similar mediation effects as in the whole MSS group, particularly regarding bullying victimization (indirect effects = 0.024, 95% CI = [0.009, 0.040], mediation effect accounted for 14.55%), as well as verbal (indirect effects = 0.057, 95% CI = [0.026, 0.095], mediation effect accounted for 15.49%), physical (indirect effects = 0.078, 95% CI = [0.041, 0.124], mediation effect accounted for 17.93%) and relational bullying (indirect effects = 0.071, 95% CI = [0.036, 0.114], mediation effect accounted for 17.07%) (Figure 1B).

In contrast, in addicted group (*n* = 281), although significant correlations were found between SVA and bullying victimization (and its subdimensions) (all *ps* < 0.007), no mediation effects of NA were observed in the association between bullying victimization and SVA.

### 3.4 NA Mediated the Relationship Between BV and SVA in CS

**FIGURE 2:**
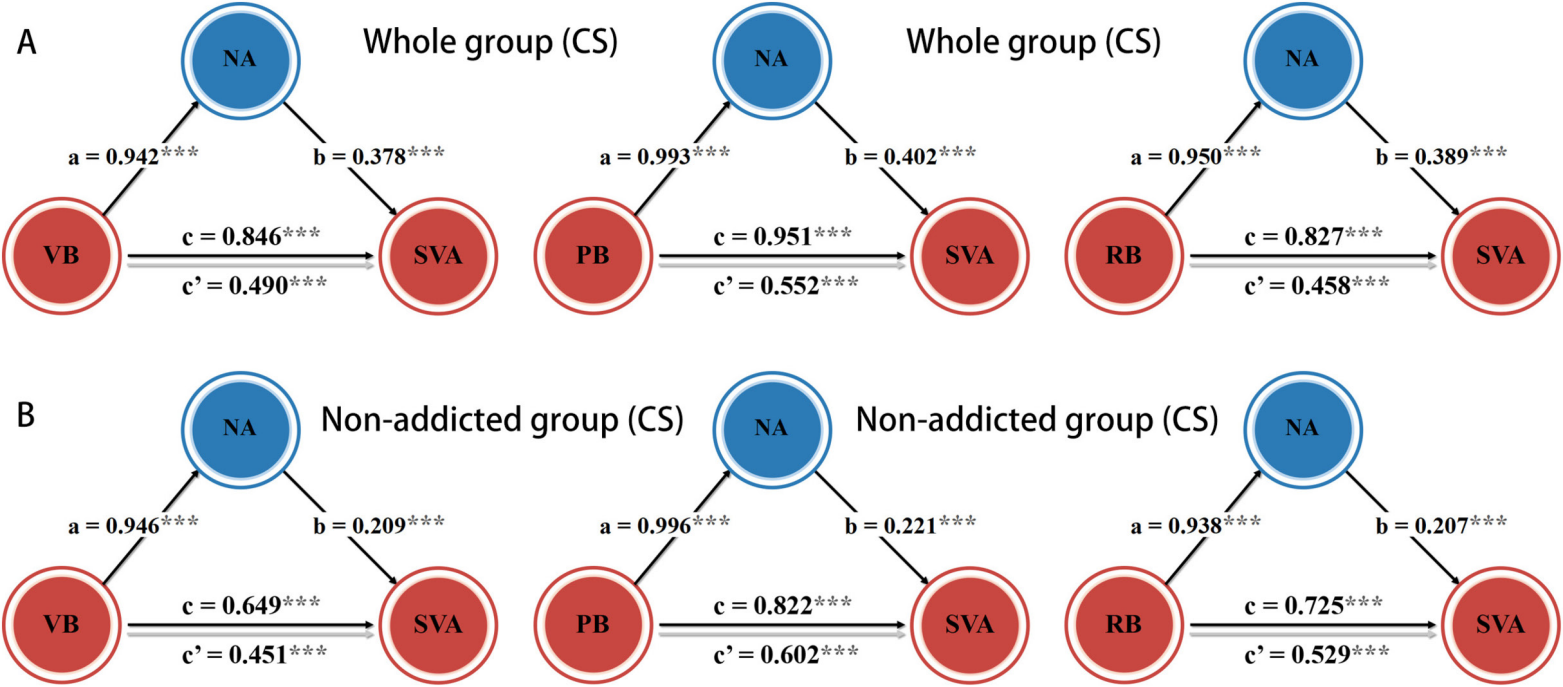
NA mediated the associations between bullying victimization and SVA in CS. The impacts of various subdimensions of bullying victimization on SVA were mediated by the NA in the whole sample of CS (*n* = 1615; A) and nonaddicted subgroup of CS (*n* = 1279; B). Abbreviations: CS, college students; NA, negative affect; PB, physical bullying; RB, relational bullying; SVA, short‐video addiction; VB, verbal bullying.

In the CS group, we observed results similar to those found in the MSS group. In particular, NA mediated the impact of bullying victimization on SVA (indirect effects = 0.128, 95% CI = [0.101, 0.158], mediation effect accounted for 39.02%). Furthermore, NA also mediated the associations between SVA and the subdimensions of bullying victimization (indirect effects = [0.356, 0.399]) (Figure 2A). In the nonaddicted group (*n* = 1279), the mediation effects of NA on the associations between SVA and bullying victimization, as well as its subdimensions, were still present (indirect effects = [0.196, 0.20]) (Figure 2B). However, in the addicted group (*n* = 336), no association was found between bullying victimization and SVA (all *ps* > 0.213), although a significant correlation between NA and SVA was observed (*r* = 0.144, *p* = 0.008).

### 3.5 Spontaneous Functional Characteristics Underlying SVA

**FIGURE 3:**
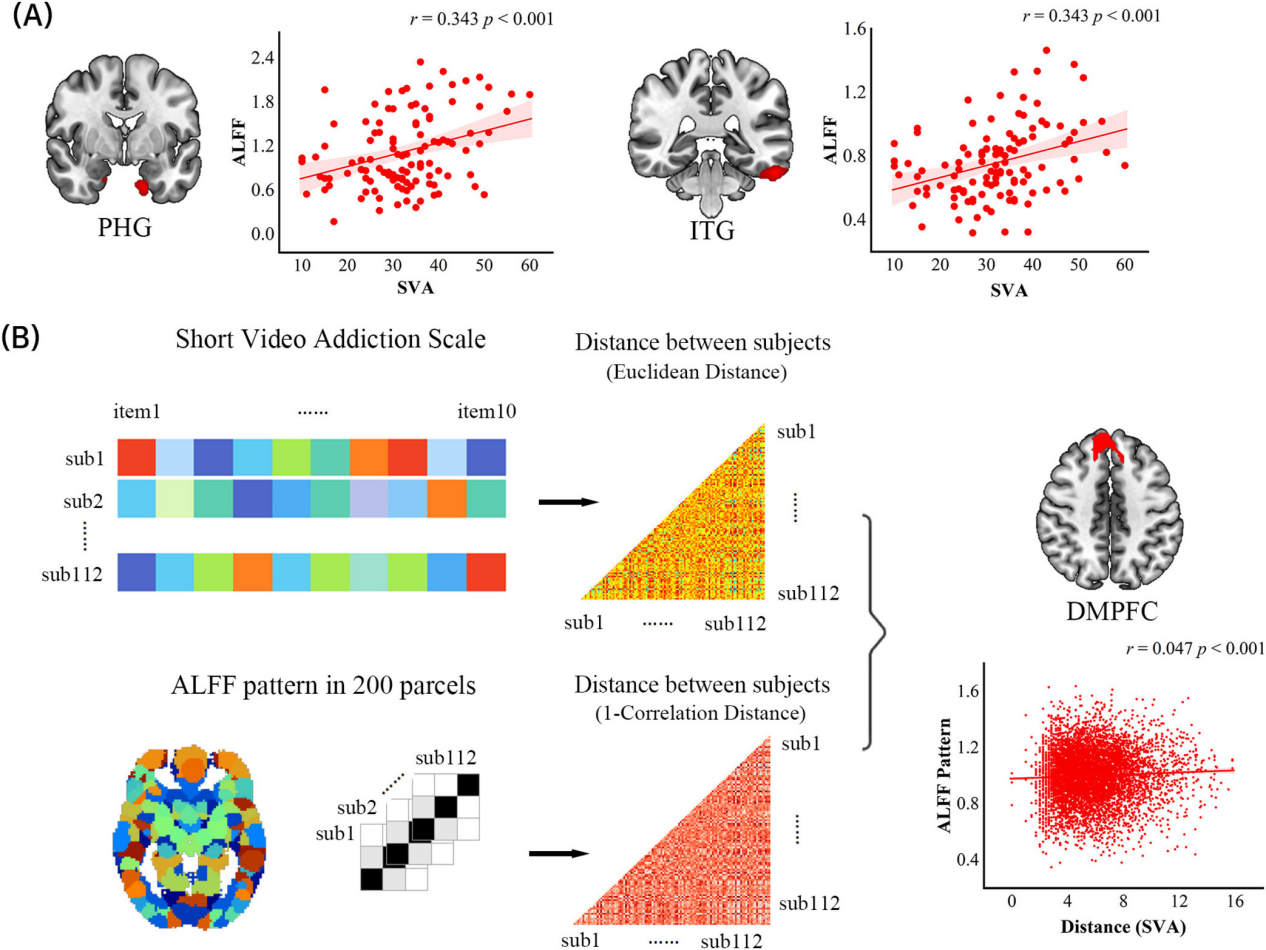
Spontaneous neural activity strength and pattern supporting SVA. SVA was positively correlated with ALFF strength in the parahippocampal gyrus (PHG) and inferior temporal gyrus (ITG) and their scatter plots were displayed (A). Intersubject representational similarity analysis (IS‐RSA) was illustrated (B), where behavioural dissimilarity and brain dissimilarity were calculated based on Euclidean distance algorithm and Pearson's correlational approach. Then, the correlations between each parcel (*n* = 200) dissimilarity matric and behavioural dissimilarity matrix were further computed via using Pearson's correlation on the lower triangle of the matrices. IS‐RSA revealed that intersubject variations in SVA were associated with ALFF patterns in the dorsomedial prefrontal cortex (DMPFC). Abbreviation: ALFF, amplitude of low‐frequency fluctuation.

The findings revealed that SVA was positively correlated with ALFF in the inferior temporal gyrus (ITG; MNI *xyz* = −56, −36, −26, Z = 4.68; cluster size = 496) and parahippocampus (PHG; xyz = −16, −2, −36, Z = 4.30; cluster size = 592) in the replicated sample (RCS; *n* = 112) (Figure 3A). Regions of interest analyses (ROIs) on above‐mentioned regions further demonstrated the robust linear correlations of SVA with ALFF in the ITG (*r* = 0.343, *p* < 0.001) and PHG (*r* = 0.343, *p* < 0.001) (Figure 3A). These ROIs analyses helped to validate the reliability of our findings.

IS‐RSA analyses revealed that intersubject variations in the SVA were associated with the ALFF pattern only in the PHG (*r* = 0.028, Bonferroni corrected *p* < 0.05). At the whole‐brain level, IS‐RSA analyses identified two additional brain regions—namely, the anterior dorsomedial prefrontal cortex (aDMPFC; *r* = 0.047, Bonferroni corrected *p* < 0.001) and posterior DMPFC (*r* = 0.047, Bonferroni corrected *p* < 0.001)—whose spontaneous brain activity patterns were significantly associated with intersubject variations in SVA (Figure 3B).

## 4 Discussion

**FIGURE 4:**
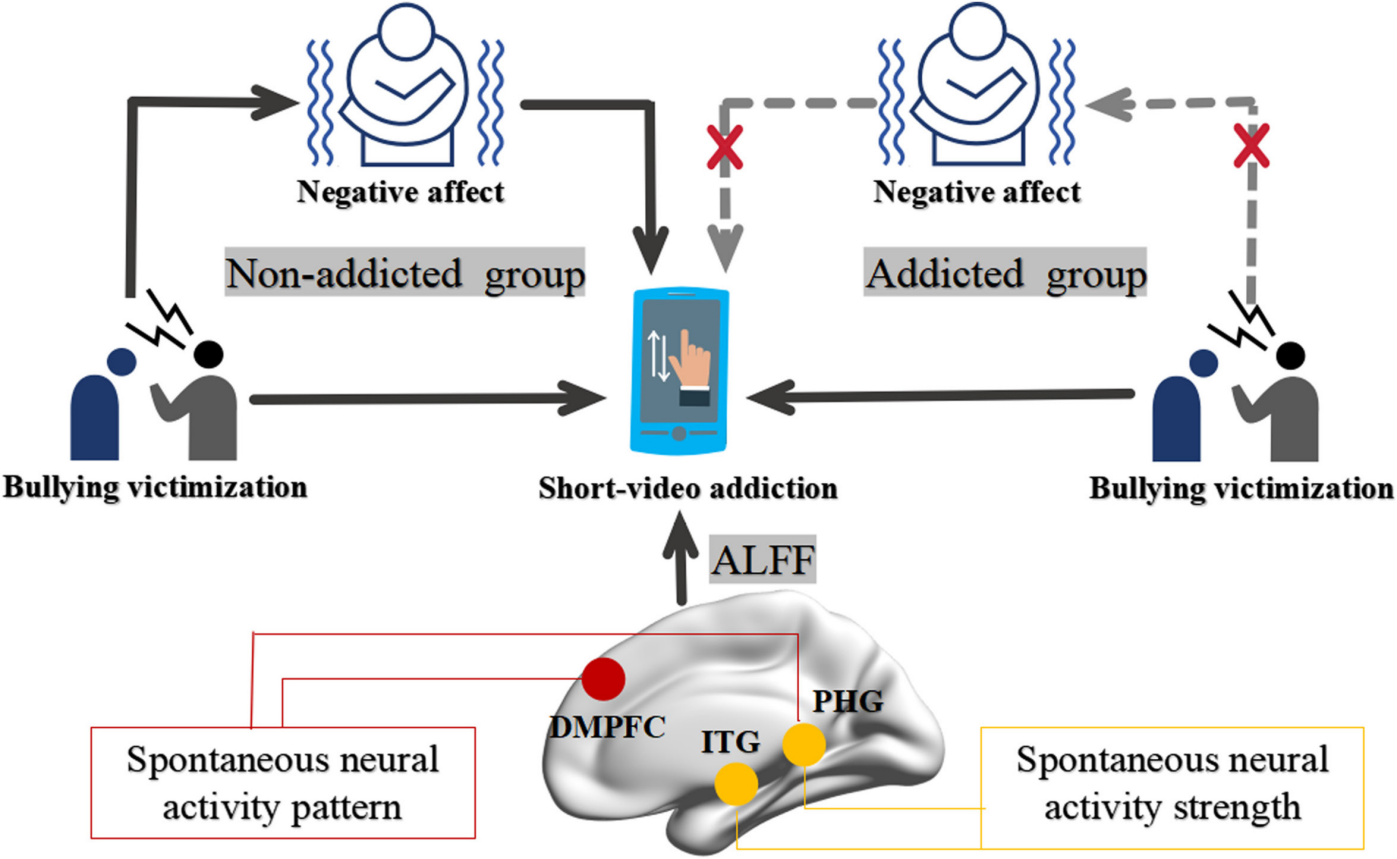
Specific mediation pathways of negative affect on the association between bullying victimization and short‐video addiction. Solid lines indicate significant correlations/mediation effects, whereas dashed lines represent diminished mediation effects. Individuals with higher short‐video addiction exhibited specific spontaneous neural activity strength and patterns in distinct brain regions.

This study systematically examined the relationship between bullying victimization (BV) and short video addiction, emphasizing the mediating role of negative affect and developmental differences across middle school and college students in a relatively large sample. Additionally, neuroimaging data from a replicated college sample (RCS) provided valuable insights into the neural correlate of SVA especially focusing on the spontaneous brain activity supporting SVA. These findings elucidate the psychological and neurobiological mechanisms underlying SVA, contributing meaningfully to the literature on behavioural addictions and relevant theory (Figure 4).

### 4.1 The Role of Bullying Victimization and Emotional Pathways in SVA

The findings highlight bullying victimization as a significant predictor of SVA, mediated partially by NA. These results align with studies on behavioural addictions, such as problematic Internet use [12] and smartphone addiction [40]. Consistent with the I‐PACE model [41], bullying victimization creates emotional distress that predisposes individuals to addictive behaviours as a maladaptive coping mechanism. The mediation effects of negative affect were stronger for verbal and physical bullying, indicating that more explicit forms of victimization may evoke heightened emotional responses. Such findings are consistent with Zhao and Zhou [2], who demonstrated that stress from adverse events intensifies emotional dysregulation, fuelling addictive tendencies in social media use.

Interestingly, the absence of negative affect's mediation effects in the addicted group suggests a shift in the underlying mechanisms driving SVA at advanced stages. In earlier stages, emotional distress from bullying victimization plays a central role, with individuals turning to short videos as a coping mechanism to alleviate negative affect. However, as addiction progresses, this reliance on emotional regulation diminishes, giving way to habitual and cue‐driven behaviours. The transition aligns with the dual‐process theory of addiction, where behaviours evolve from goal‐directed, emotionally motivated actions to automatic, habit‐based responses [42].

The discrepancy in mediation patterns between nonaddicted and addicted groups can be attributed to the developmental nature of social compensation theory. For nonaddicted individuals, the heightened social media use among bullying victims mediated by NA aligns with the theory, as it reflects the conscious use of media as an emotion regulation strategy to alleviate psychosocial stress [43]. However, the absence of NA‐mediated effects among addicted users signifies a fundamental shift in compensatory mechanisms. The shift signals a transition from instrumental compensation (using media for emotion regulation) to habitual compensation (a habitual pattern of behaviour). This developmental change is consistent with the theory of compensatory Internet use [8], which posits that recurrent consumption behaviours, triggered stressors, become autonomously reinforced by environmental cues (e.g., algorithmic recommendations) and operant conditioning, gradually decreasing reliance on emotional motives.

The I‐PACE model [41] further explains this shift, suggesting that as addiction develops, cognitive‐emotional dispositions interact with reinforcement mechanisms, transforming compensatory media use from a conscious coping strategy to an automatic behaviour sustained by a stimulus–response cycle rather than emotional regulation. The disappearance of mediation effects does not contradict the theory but rather signifies an evolutionary shift in the compensatory process—from conscious emotion regulation to behavioural automation triggered by environmental cues.

The disappearance of negative affect's mediation effect underscores the need for stage‐specific interventions. For individuals in the addicted group, strategies targeting emotional distress may be less effective. Instead, approaches focused on breaking habitual cycles, such as cue exposure therapy or interventions enhancing cognitive control [44], may yield more promising outcomes. These findings emphasize the evolving nature of SVA and the importance of tailoring intervention strategies to the stage of addiction.

### 4.2 Developmental Differences in the BV–SVA Relationship

The developmental differences in the BV–SVA relationship were closely tied to distinct neurodevelopmental characteristics. Adolescents, represented by the middle school student (MSS) group, showed weaker associations between bullying victimization, negative affect and SVA compared to college students (CS). During adolescence, the limbic system, particularly the ventral striatum and amygdala, exhibits heightened activity in response to rewarding stimuli, whereas the prefrontal cortex responsible for self‐regulation continues to mature into the mid‐20s [17]. This imbalance between heightened reward sensitivity and immature cognitive control makes adolescents particularly vulnerable to SVA, especially when distress from bullying victimization is present. The emotional reactivity of adolescents, driven by increased amygdala activity, further exacerbates their reliance on short video consumption as a coping mechanism for negative emotions.

Given these vulnerabilities, adolescence represents a critical intervention window for preventing or mitigating the development of SVA. The heightened neuroplasticity during this period offers opportunities for targeted interventions aimed at strengthening self‐regulation and emotional coping strategies [45]. Programmes designed to enhance prefrontal cortex function through activities like mindfulness training, physical exercise and cognitive‐behavioural therapy (CBT) could improve adolescents' ability to manage distress without resorting to maladaptive coping mechanisms such as excessive video consumption. Furthermore, educating adolescents about the manipulative aspects of personalized video recommendations and fostering digital literacy skills may help reduce their susceptibility to compulsive engagement with short video platforms.

In young adults, represented by the CS group, the stronger associations between bullying victimization, negative affect and SVA reflect the neurodevelopmental stability characteristic of early adulthood. The contribution from bullying to reshape the PFC and its functional connectivity with the limbic system gradually disrupt the emotional regulation and impulse control [46], heightening the impact of bullying victimization on negative affect and subsequent SVA. Furthermore, the diminished sensitivity of the reward system in young adults likely facilitates their compulsive engagement with short videos, which rely heavily on immediate gratification [47, 48, 49]. However, intervention strategies targeting habitual behaviours may still be effective in this group, especially those addressing environmental triggers and reinforcing alternative, healthier coping mechanisms.

The distinct developmental trajectories observed between adolescents and young adults underscore the importance of tailoring interventions to the neurobiological and psychosocial characteristics of each group. Early prevention efforts focused on adolescents, in particular, hold the greatest promise for curbing the long‐term consequences of SVA, given their heightened neuroplasticity and ongoing brain development.

### 4.3 Neural Mechanisms Underlying SVA

The neuroimaging findings align with recent research emphasizing key brain regions involved in SVA, including the DMN, SN and DAN. These neural circuits related to reward processing, attention and emotional regulation.

Personalized video recommendations modulate the DMN, particularly the DMPFC, which is involved in self‐referential processing and social cognition [18, 50]. This increased DMN activity during video consumption fosters introspection and sustained engagement, reinforcing attentional focus and content relevance. The coactivation of the DMN and reward regions, such as the VTA, further strengthens video consumption by triggering dopaminergic pathways linked to pleasure and reward [18]. Additionally, functional connectivity between the SN and other networks is crucial for detecting engaging content, with the anterior insula and anterior cingulate cortex shifting attention to salient stimuli, thus impairing self‐regulation [50]. The integration of the DAN with the DMN and SN further supports sustained attention, highlighting the power of short video platforms in capturing attention and fostering compulsive behaviours. Repeated activation of these networks leads to neuroplastic changes, including reduced grey matter volume in prefrontal and temporal regions in individuals with excessive smartphone use [50].

Furthermore, findings on the amplitude of low‐frequency fluctuation (ALFF) provide additional insights into the neural mechanisms underlying SVA. ALFF measures spontaneous neural activity and reflects the intensity of regional brain activity at rest [38]. The increased ALFF observed in regions such as the ITG and PHG suggests heightened baseline activity in areas associated with visual processing, memory and emotional regulation during short video consumption. These regions may contribute to the immersive and emotionally engaging experience of personalized content, reinforcing habitual behaviours. Moreover, alterations in ALFF within the dMPFC and HPG highlight its role in self‐referential processing and emotional memory [51, 52], further linking these spontaneous fluctuations to the compulsive use patterns characteristic of SVA.

Taken together, these findings collectively indicate that SVA is deeply rooted in the interaction of neural circuits responsible for reward processing, emotional regulation, attentional focus and spontaneous neural activity. Understanding these mechanisms offers valuable insights into the neurobiological basis of SVA and its reinforcement through personalized content delivery.

### 4.4 Limitations and Future Directions

Despite its strengths, this study has limitations. The cross‐sectional design precludes causal inferences, and the absence of neuroimaging data for adolescents limits the generalizability of the neural findings to younger populations. Additionally, although the involvement of regions like the PHG, ITG, DMPFC and ALFF provides valuable insights, the interpretation of these findings remains constrained by the broad functional roles of these regions and measures. Further studies using task‐based neuroimaging paradigms are needed to disentangle these functions and clarify the causal mechanisms linking these regions to SVA. Longitudinal research could also illuminate the dynamic interplay between these brain regions and the progression of SVA, providing a more comprehensive understanding of the neural basis of this behavioural addiction.

## 5 Conclusion

In conclusion, this study underscores the complex interplay of bullying victimization, emotional distress and neural mechanisms in shaping short video addiction. By addressing these psychosocial and neurobiological factors, targeted interventions can mitigate addiction risk, particularly in vulnerable populations. These findings contribute to the growing body of research on the psychological and neural underpinnings of behavioural addictions, offering directions for both prevention and treatment.

## Author Contributions

*Conceptualization*: Qiang Wang. *Methodology*: Qiong Yao and Yuanyuan Gao. *Investigation*: Qiong Yao, Jinlian Wang and Chang Liu. *Analysis*: Qiong Yao and Yuanyuan Gao. *Writing*: Qiong Yao, Qiang Wang, and Wenwei Zhu. *Supervision*: Qiang Wang and Guang Zhao.

## Conflicts of Interest

The authors declare no conflicts of interest.

## Data Availability

The data that support the findings of this study are available on request from the corresponding author. The data are not publicly available due to privacy or ethical restrictions.
